# Disruption of mitochondrial function in blood and sexual stages of *Plasmodium falciparum* by ferulenol

**DOI:** 10.1186/s13071-025-07103-4

**Published:** 2025-11-05

**Authors:** Gamolthip Niramolyanun, Chonnipa Praikongkatham, Wang Nguitragool, Jetsumon Sattabongkot, Niwat Kangwanrangsan

**Affiliations:** 1 Department of Pathobiology, Faculty of Science, Mahidol University, Bangkok, Thailand; 2 Mahidol Vivax Research Unit, Faculty of Tropical Medicine, Mahidol University, Bangkok, Thailand; 3 Department of Molecular Tropical Medicine and Genetics, Faculty of Tropical Medicine, Mahidol University, Bangkok, Thailand

**Keywords:** Antimalarial drug, Ferulenol, Gametocytes, Mitochondria, Falciparum malaria, Transmission-blocking agent

## Abstract

**Background:**

Malaria continues to be a significant global health challenge, necessitating the development of novel antimalarial compounds. This study explores the effects of ferulenol on multiple lifecycle stages of *Plasmodium falciparum*. Ferulenol has been identified as a promising antimalarial candidate, demonstrating high efficacy in inhibiting asexual blood-stage parasites at low micromolar concentrations. However, its effects on other parasite stages remain unexplored, despite its mitochondrial target being critical for sexual stage development. This study aims to investigate ferulenol’s potential as a dual-target antimalarial by assessing its effects on development of asexual blood-stage and transmission precursor-stage parasites.

**Methods:**

Falciparum malaria parasites were cultured in vitro and incubated with or without ferulenol. Effects of the treatment on the development of the asexual blood-stage, early-stage gametocyte, late-stage gametocyte, and gamete formation were assessed using light microscopy. The impact of ferulenol on mitochondrial membrane potential was investigated using JC-1 staining and analyzed by fluorescence microscopy.

**Results:**

The highest dose of ferulenol inhibited asexual blood-stage proliferation by 88%, early-stage gametocyte development by 82%, and stage V gametocyte maturation at about 90%. Moreover, the effect of ferulenol was more pronounced on male gamete formation than on female gamete formation, with the development inhibited at 81% and 27%, respectively.

**Conclusions:**

These findings position ferulenol as a promising dual antimalarial activity on asexual blood-stage and gametocyte stages, which could lead the compound to disrupt both severity and transmission of disease.

**Graphical Abstract:**

**Figure:**
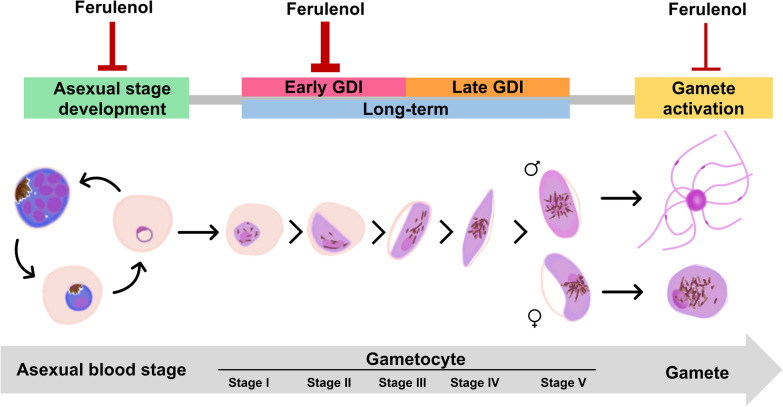

**Supplementary Information:**

The online version contains supplementary material available at 10.1186/s13071-025-07103-4.

## Background

Malaria is an ancient disease that has been recognized for over a century, yet it continues to pose a significant global health burden [1]. Each year, malaria causes hundreds of thousands of deaths, with children under the age of 5 being particularly vulnerable [2]. Although several antimalarial drug classes have been developed for the treatment and control of the disease, the emergence of drug resistance remains a serious challenge in many endemic areas. Several studies attempted to discover and develop new antimalarial drugs that target essential biological pathways involved in parasite survival [3, 4]. One important target was the mitochondrion, the powerhouse responsible for ATP production. Although previous studies showed that ATP generated through glycolysis alone was sufficient for the parasite’s survival during the asexual blood stage [5–9], the mitochondrion remained an attractive target for drug development due to its additional, non-ATP-related function. In addition, mitochondrial activity became increasingly important during the parasite’s sexual stages. Previous studies have reported elevated mitochondrial activity and ATP demand during gametocyte development [9–12], supported by ultrastructural changes in the mitochondria and genetic evidence highlighting their essential role in sexual stage progression [10].

One good example was atovaquone, a recent clinically approved antimalarial, which targeted the mitochondrial electron transport chain (ETC) [13–15] and exhibited activity against both the asexual blood stages and gametocytes of *Plasmodium falciparum* [16–18]. Its success underscores the therapeutic potential of mitochondrial inhibitors and blocks both parasite replication and transmission.

Ferulenol, a sesquiterpene prenylated coumarin derivative, had been identified as a potential antimalarial compound targeting mitochondrial function. It had been shown to inhibit malate:quinone oxidoreductase (MQO), an enzyme involved in the TCA cycle, as well as the *bc*_1_ complex, a critical element of the mitochondrial ETC [19–22]. Ferulenol demonstrated inhibitory effects against the asexual blood stage at low micromolar concentrations. However, its effect on the parasite’s sexual stages, including gametocyte development and gamete formation, remained unexplored.

This study aimed to investigate the effects of ferulenol on *P. falciparum* development across multiple stages of the life cycle, including the asexual blood stage, gametocyte, and gamete. By elucidating its activity in these stages, we seek to provide foundational evidence for the development of ferulenol as a dual-target antimalarial drug with the potential to disrupt both parasite proliferation and transmission.

## Methods

### In vitro culture of *P. falciparum* asexual and gametocyte stages

*Plasmodium falciparum* NF54, a gametocyte-producing strain, was cultured in a medium composed of RPMI 1640 with l-glutamine (Gibco), supplemented with 2 g/l sodium bicarbonate (Mallinckrodt), 25 mM HEPES (Sigma), 2 g/l dextrose (M&B), 50 mg/l hypoxanthine (Sigma), 10% heat-inactivated human serum type A positive, and 10 mg/l gentamicin (Siam Pharmaceutical) [23]. The culture was maintained with daily medium changes at 5% hematocrit in a T75 flask, incubated at 37 ºC with a 90% N_2_, 5% O_2_, and 5% CO_2_ gas mixture. Parasite development was monitored daily using Giemsa-stained thin blood smears to assess the percentage of parasite, developmental stage, and morphology under the Nikon Eclipse Ni-U upright microscope (Nikon).

In vitro induction of *P. falciparum* gametocytes was performed using a modified procedure based on Bounkeua et al. [24]. Briefly, a synchronized asexual-stage culture was cultivated to 3–5% parasitemia and then diluted to 1% at the ring stage in 15 ml of complete medium (CM) without antibiotics. This was counted as day 1 of gametocyte culture. Daily medium replacement was performed until the parasitemia returned to 3–5%. The culture was subsequently maintained at 25 ml for 10–12 days with 4% hematocrit, and the medium was replaced daily. To prevent premature gamete formation, the culture was maintained on a 37 °C warmer plate during each medium change before incubation in a hypoxic chamber containing mixed gases at 37 °C.

### Development inhibition assay

**Fig. 1:**
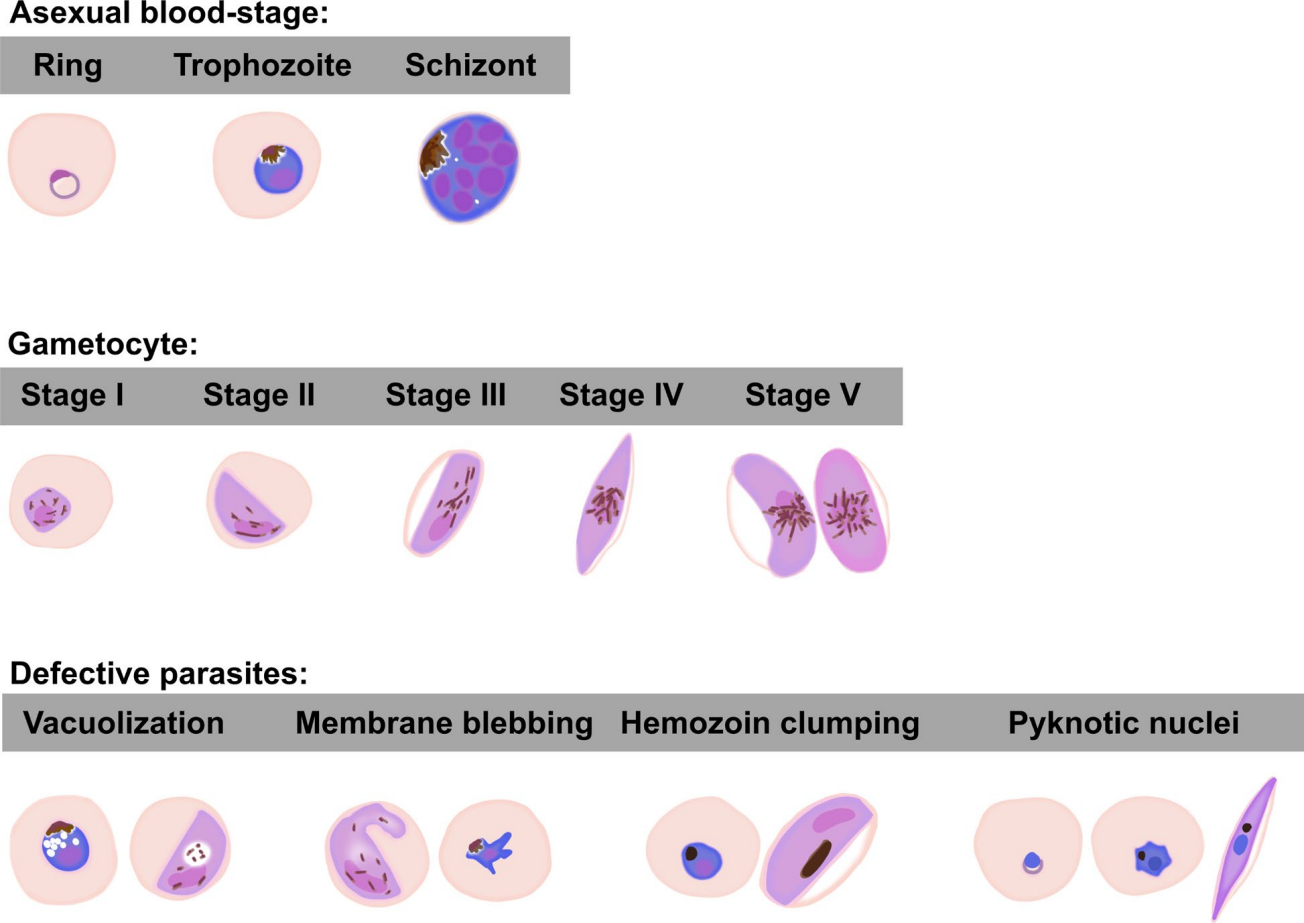
Morphological classification of *Plasmodium falciparum* blood-stage parasites and representative defective parasites. The top panel shows the three asexual stages—ring, trophozoite, and schizont—based on nuclear and hemozoin features. The middle panel shows five stages of gametocyte maturation (stages I–V), characterized by changes in shape, nuclear character, and hemozoin distribution. The bottom panel demonstrates representative morphological defects observed in both asexual and gametocyte stages, including vacuolization, membrane blebbing, hemozoin clumping, and pyknotic nuclei. These criteria were used to stage parasites and identify abnormal forms in this study

A synchronized culture of 0.3% ring-stage parasitemia was treated with ferulenol at various concentrations ranging from 0.01 to 100 µM, alongside a vehicle control (0.1% DMSO), for 72 h in a 96-well plate. Daily medium changes were performed. To examine the temporal dynamics of drug action, samples were harvested every 24 h (24, 48, and 72 h), and Giemsa-stained thin blood smears of each condition were prepared to investigate developmental inhibition and morphological abnormalities under the light microscope, using the criteria specified in Fig. 1. Experiments were conducted using three biological replicates, each with two technical replicates.

### Gametocyte development inhibition (GDI) assay

The assay was performed following Adjalley’s protocol with modifications [25]. Gametocyte development inhibition was assessed across two treatment periods: early gametocyte development inhibition (early GDI) and late gametocyte development inhibition (late GDI). Gametocyte cultures at 4% hematocrit were treated with ferulenol or 0.1% DMSO (vehicle control) for 3 consecutive days with daily medium changes. The treatment started on Day 3 (stage I, II) for early GDI and on Day 7 (stage III) for late GDI. After the treatment periods, samples were harvested, and Giemsa-stained blood smears were prepared to investigate development inhibition and morphological defects according to the criteria in Fig. 1. A total of 10,000 erythrocytes were examined per sample under a light microscope. Each experiment included three biological replicates, each comprising two technical replicates. The formula to calculate %inhibition is:

$$\% {\text{Inhibition}}\,{ = }\,{100}\, - \,\left( {\frac{{\% {\text{parasitemia/}}\% {\text{gametocytemia of treated group}}}}{{\% {\text{parasitemia/}}\% {\text{gametocytemia of untreated control}}}}\, \times \,{100}} \right)$$

### Gamete activation

To investigate the effect of ferulenol on gamete formation, stage V gametocytes (on Day 13 of gametocyte culture) were treated with ferulenol at concentrations of 1, 10, and 100 µM for 24 h. On the following day, 200 µl of gametocyte culture was spun down to remove the supernatant, and 100 µl of exflagellation medium containing 100 µM xanthurenic acid (21 °C pre-cooled) was added. After incubation at room temperature for 20 min, exflagellation centers were counted using a hemocytometer, and blood smears were prepared to examine the morphology and number of macrogametes formed [26, 27].

### Mitochondrial membrane potential

JC-1 is a fluorescent lipophilic carbocyanine dye used to assess mitochondrial membrane potential. Following ferulenol treatment, parasites were harvested, washed with incomplete medium (ICM), and stained with 5 µM JC-1 for 30 min at 37 °C. After being washed twice with 1 × PBS, > 20 parasites in each condition were evaluated [28]. Images of parasite mitochondria emitting red and green fluorescence were captured using a Nikon Eclipse Ni-U upright microscope. NIS-Elements BR software (Nikon) was used to measure the intensity of red and green fluorescence. The ratio of red to green fluorescence in each condition was calculated and used to compare mitochondrial membrane potential across groups.

### Statistical analysis

All statistical analyses were performed using GraphPad Prism. Significant differences between treatment groups were assessed using one-way analysis of variance (ANOVA), followed by Tukey's multiple comparison test. The half-maximal inhibitory concentration (IC₅₀) values were determined using nonlinear regression analysis with a log(inhibitor) versus normalized response variable slope model. Data are presented as mean ± standard deviation (SD), and *P* < 0.05 were considered statistically significant.

## Results

### Ferulenol impaired the development and survival of asexual blood-stage *P. falciparum*

The mitochondrion is often referred to as the powerhouse of the cell because of its role in producing the energy necessary for cellular activities in eukaryotic cells. However, some studies suggested that the mitochondrial energy metabolism in malaria parasites might not be essential for the development of the asexual stage of the malaria parasite [9]. To investigate whether ferulenol, a mitochondrial ATP synthesis inhibitor, could interrupt the development of the asexual blood-stage parasite *P. falciparum*, an NF54 line was utilized for this study.

**Fig. 2:**
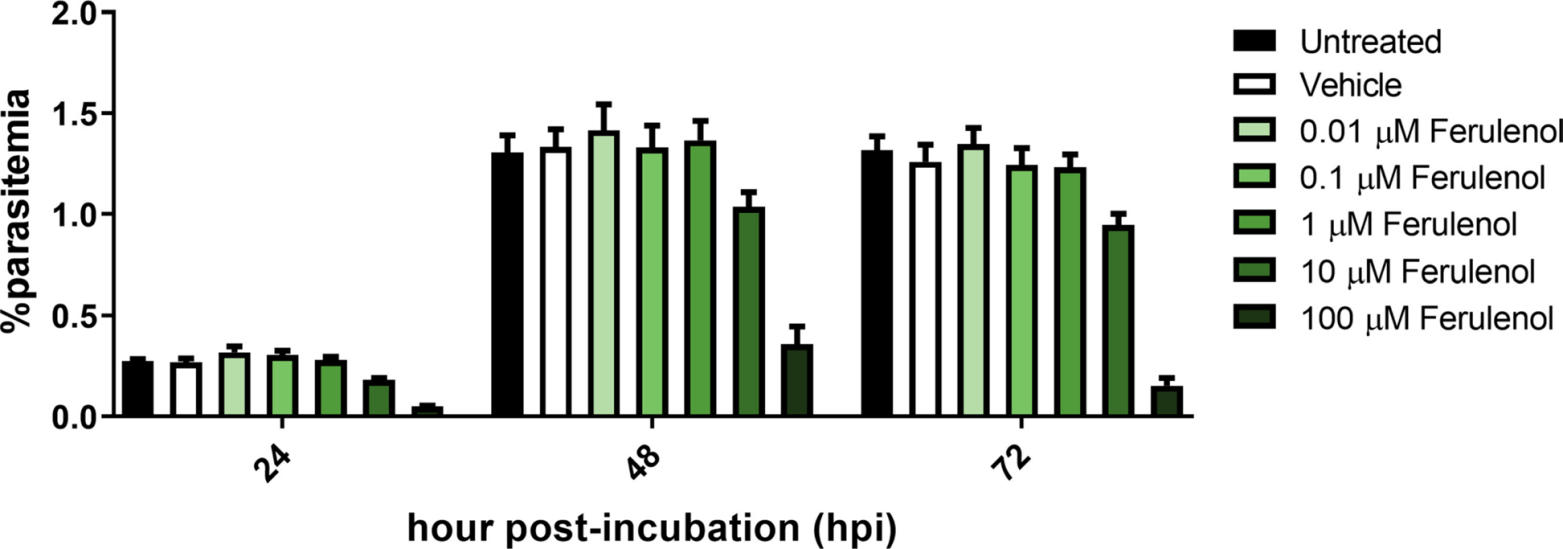
Dose-dependent inhibition of parasite growth by ferulenol. The bar graph shows the percentage of parasitemia at 24-, 48-, and 72-h post-incubation (hpi) under untreated, vehicle control (0.1%DMSO), and ferulenol-treated conditions at varying concentrations (0.01, 0.1, 1, 10, and 100 µM). The data indicate a dose-dependent effect of ferulenol, with higher concentrations leading to greater growth inhibition at all time points. Experiments were conducted using three biological replicates, each with two technical replicates. Error bars represented the mean ± SD from three independent experiments

**Fig. 3:**
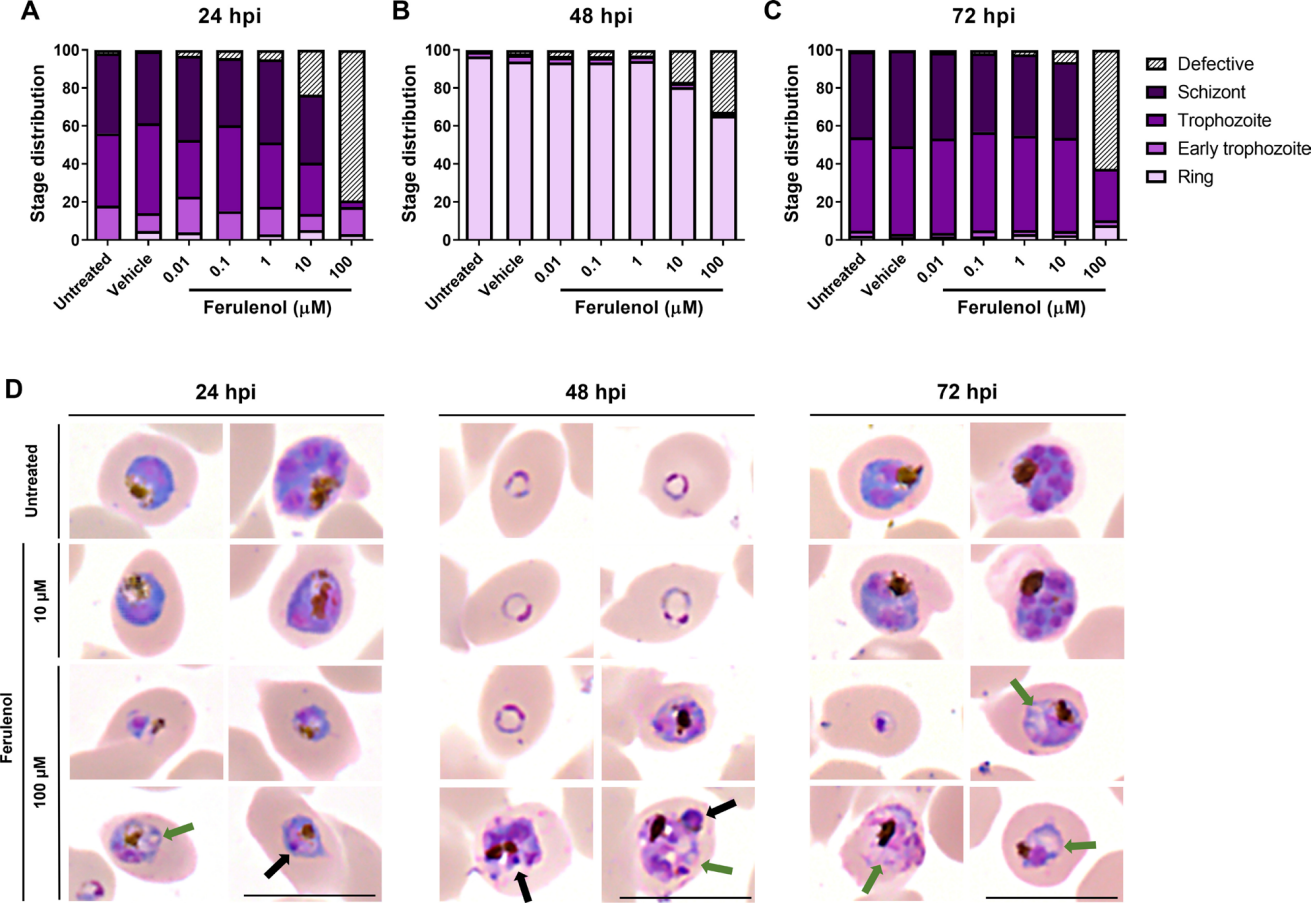
Stage distribution and morphological effects of ferulenol treatment on *Plasmodium falciparum* asexual blood stage. (**A**–**C**) Bar charts represent the distribution of parasite developmental stages (rings, early trophozoite, trophozoites, schizonts, and defective parasite) at 24-, 48-, and 72-h post-incubation (hpi) under untreated, vehicle control, and ferulenol treatment conditions. Data show dose-dependent arrest of parasites at the ring and trophozoite stages upon ferulenol treatment. (**D**) Representative Giemsa-stained images of parasites at 24, 48, and 72 hpi under different treatment conditions are illustrated. Untreated and vehicle controls display normal morphology, while ferulenol-treated parasites exhibit significant morphological changes, including cytoplasmic vacuolization (green arrows) and nuclear abnormalities (black arrows). Experiments were conducted using three biological replicates, each with two technical replicates. Scale bars indicated 10 µm

At 24 h post-incubation (hpi), parasites in the untreated and vehicle control conditions developed normally into trophozoites and schizonts, maintaining consistent percentages of parasitemia. In contrast, treatment with ferulenol inhibited parasite development in a dose-dependent manner (Fig. 2). At a concentration of 100 µM, ferulenol inhibited parasite growth by 82.0 ± 4.0% and induced pathological changes that impaired parasite survival. Additionally, residual parasites exhibited growth retardation, remaining in the early trophozoite and trophozoite stages (Fig. 3A, D).

By 48 hpi, normal parasites in all conditions were able to egress and invade new red blood cells, generating the ring stage. Even under the highest ferulenol concentration, the reduced parasite numbers observed at 24 hpi rebounded by 48 hpi, resulting in overall growth inhibition of 72.7 ± 16.7% at 48 hpi (Fig. 2). This suggests that the surviving parasites could progress to the schizont stage and invade new red blood cells (Fig. 3B, D).

At the final time point (72 hpi), parasites in the untreated and vehicle control groups had progressed to the trophozoite and schizont stages. In the 100 µM ferulenol treatment group, an 88.4 ± 7.0% reduction in parasite count was observed (Fig. 2), with most remaining parasites arrested at the ring and trophozoite stages (Fig. 3C, D). This indicates that a high dose of ferulenol inhibited the development of asexual blood-stage parasites.

Notable morphological changes in ferulenol-treated parasites were evident as early as 24 hpi, affecting both the cytoplasm (Fig. 3D; green arrow) and the nucleus (Fig. 3D; black arrow). Cytoplasmic defects were characterized by the formation of numerous vacuoles, giving the cytoplasm a "bubbled" appearance. Nuclear abnormalities indicative of cell death processes were also observed, including pyknotic nuclei and karyorrhexis, which is an irreversible injury process of the cell.

### Ferulenol is a potential inhibitor of *P. falciparum* gametocyte development and maturation

**Fig. 4:**
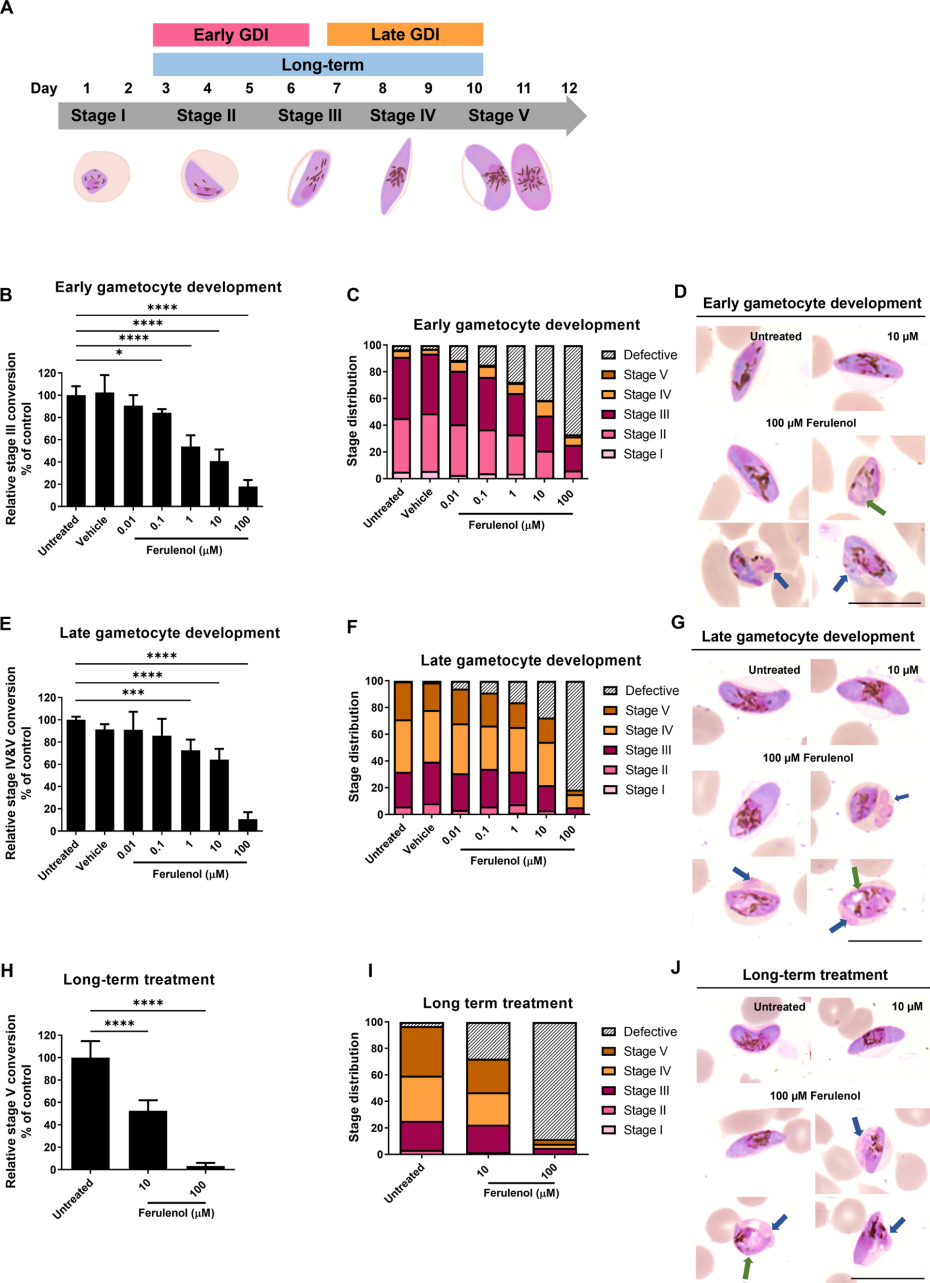
Effects of ferulenol on gametocyte development in *Plasmodium falciparum*. **A** Schematic representation of the ferulenol treatment strategy, showing the duration of drug exposure for early gametocyte development inhibition (EGDI), late gametocyte development inhibition (LGDI), and long-term treatment according to the stage of gametocyte development. **B** The bar graph represents the dose-dependent inhibition of early-stage gametocyte development by reducing the transition from stages I/II to stage III gametocyte in a dose-dependent manner. **C** The stacked bar chart shows gametocyte stage distribution on Day 6. **D**, **G**, and **J** Representative Giemsa-stained images of gametocytes treated with ferulenol compared to untreated controls are illustrated. Morphological defects in gametocytes, including cytoplasmic vacuolization (green arrow) and plasma membrane irregularities (blue arrow), are indicated. **E** Bar chart presented significant inhibition of late-stage gametocyte transitions (stages III to IV/V), particularly at 100 µM ferulenol. **F** The stacked bar chart shows the proportional distribution of gametocyte stage within each treatment condition on Day 10. **H** The bar graph demonstrates that long-term ferulenol treatment inhibits the progression of early-stage gametocytes to stage V gametocytes in a dose-dependent manner. **I** The stacked bar chart represents gametocyte stage distribution after long-term treatment with ferulenol. All scale bars indicate 10 µm. Results are presented as mean ± SD from three independent experiments, each with two technical replicates. Statistical significance was assessed using one-way ANOVA (*P < 0.05; ***P < 0.001; ****P < 0.0001)

Gametocytes are the precursors of the sexual stage responsible for malaria transmission. Among *Plasmodium* species, *P. falciparum* uniquely requires five morphologically distinct developmental stages to produce mature stage V gametocytes, which are capable of transmitting to mosquitoes. To investigate the inhibitory effects of ferulenol on gametocyte development (Fig. 4A), gametocytes of the *P. falciparum* NF54 strain were categorized into two groups based on differences in morphology and cellular metabolism: early-stage gametocytes (stages I to III) and late-stage gametocytes (stages IV and V).

To examine the effect of ferulenol on early-stage gametocyte development, gametocyte cultures were treated with various concentrations of ferulenol from Day 3 to Day 6. On Day 6, parasites from each condition were harvested to evaluate gametocyte development and morphological changes. The results showed that ferulenol significantly inhibited the development of early-stage gametocytes, as evidenced by a dose-dependent decrease in the %conversion of stage I and II gametocytes to stage III compared to the untreated control (Fig. 4B). The highest dose (100 µM ferulenol) reduced early-stage gametocyte development by 82.0 ± 5.9%. Additionally, the number of stage II gametocytes in the culture decreased in a dose-dependent manner (Fig. 4C). These findings suggest that ferulenol disrupts early-stage gametocyte development.

The effect of ferulenol on late-stage gametocyte development was assessed by treating gametocyte cultures from Day 7 to Day 10. Although on Day 10 inhibitory effects were observed in a dose-dependent manner, only the 100-µM ferulenol condition achieved significant inhibition of late-stage gametocyte development, with an 89.3 ± 6.4% reduction (Fig. 4E, F). These results highlighted that ferulenol impaired late-stage gametocyte development in a dose-dependent manner. However, whether the surviving parasites retain transmission capacity was not assessed, and the implications for transmission remain to be determined.

To evaluate whether early-stage gametocytes treated with ferulenol over a long period could progress to stage V, gametocyte cultures were treated with 10 µM and 100 µM ferulenol from Day 3 to Day 10. Fresh drug and medium were prepared and replaced daily. Parasites were harvested for analysis on Day 10. The results demonstrated that ferulenol inhibited gametocyte maturation to stage V in a dose-dependent manner, reducing maturation by 47.40 ± 9.40% and 96.8 ± 2.9% with 10 µM and 100 µM ferulenol, respectively (Fig. 4H, I). This confirms the inhibitory effect of ferulenol on gametocyte development, underscoring its potential to reduce parasite transmission.

Morphological defects in gametocytes were observed in both early and late GDI experiments, affecting the cytoplasm (Fig. 4D, G, and J; green arrow) and membrane (Fig. 4D, G, and J; blue arrow). Cytoplasmic abnormalities included vacuolization, resembling the defects seen in asexual blood-stage parasites treated with ferulenol. Membrane irregularities, such as protrusions or blebbing of the parasite plasma membrane, were also observed.

### Ferulenol impairs male and female gamete formation

Mitochondrial energy requirements are known to increase during the sexual stage of the parasite [5, 9, 11]. To investigate the effect of ferulenol on gamete formation, mature gametocytes were incubated with various concentrations of ferulenol for 24 h on Day 12. The following day, parasites from each condition were harvested to assess defects in male and female gamete generation.

**Fig. 5:**
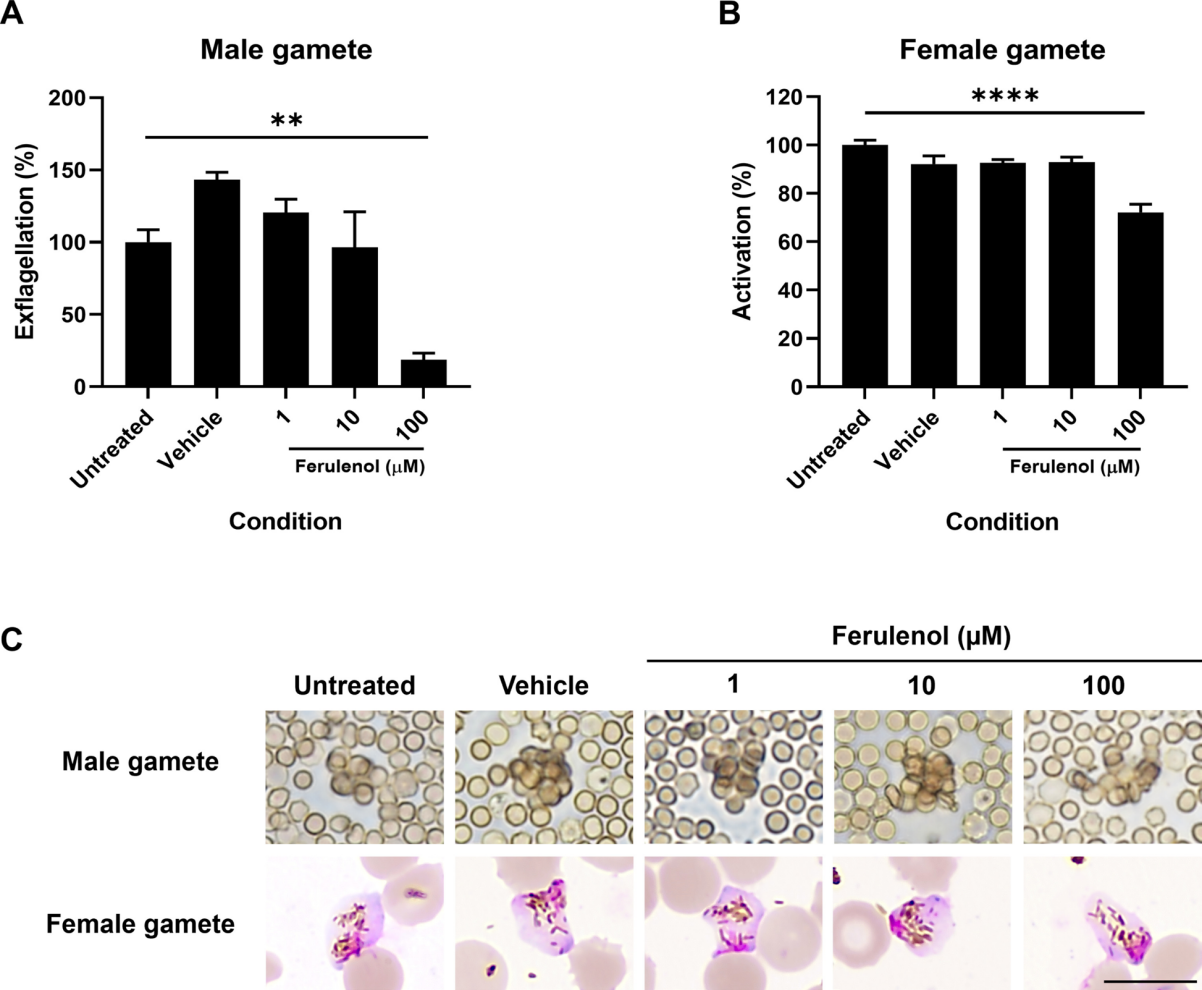
Impact of ferulenol on male and female gamete formation in *Plasmodium falciparum*. **A** The bar graph represents the percentage of exflagellation centers. Treatment with 100 µM ferulenol significantly inhibited exflagellation. **B** The bar graph shows the number of female gamete formations analyzed from Giemsa-stained blood smear after gamete activation. The highest concentration of ferulenol (100 µM) significantly reduced female gamete formation. **C** Representative images of a male exflagellation center and a Giemsa-stained female gamete are shown (scale bar = 10 µm). Results are presented as mean ± SD from two independent experiments, each with two technical replicates. Statistical significance was assessed using one-way ANOVA (**P < 0.01; ****P < 0.0001)

Male gamete (microgamete) generation was evaluated by counting the number of exflagellation centers. The results showed a significant reduction in the number of exflagellation centers in the 100 µM ferulenol treatment group. At this concentration, ferulenol inhibited the exflagellation process by up to 81.2 ± 8.9% (Fig. 5A). Female gametes develop from the crescent-shaped mature stage V gametocytes, which undergo a rounding-up process and egress from the host red blood cell. At this stage, the parasite exhibits a round shape without red blood cell membrane. The cytoplasm appears blue, while the condensed nucleus is pink in Giemsa-stained smears. Although the highest concentration of ferulenol (100 µM) inhibited female gamete formation by only 27.9 ± 6.8%, it was statistically significant (Fig. 5B). These findings suggest that mitochondrial energy metabolism, inhibited by ferulenol, was essential for the formation of both male and female gametes.

### Early-stage gametocytes are most sensitive to ferulenol

To compare the effect of ferulenol across different stages, the number of intact parasites in each condition was used to determine the half-maximal inhibitory concentration (IC_50_). The results revealed the IC_50_ values in ascending order as follows: early-stage gametocyte (3.34 µM), late-stage gametocyte (11.92 µM), asexual blood stage (21.67 µM), male gamete exflagellation (50.09 µM), and female gamete formation (> 100 µM) (Table 1). The IC_50_ result indicated that early-stage gametocyte development could be interrupted by ferulenol at the lowest concentration compared to the other stages. Comparing the precursors of the sexual stage (gametocytes) and the asexual stage, it could be inferred that gametocytes were more susceptible to ferulenol than asexual stages. Additionally, male gametes appeared to be more sensitive to ferulenol than female gametes.

**Table 1:** Summary of the half maximal inhibitory concentration (IC_50_)

Parasite stages	IC_50_ value^a^ (µM)
Asexual blood stage	21.67
Early-stage gametocyte	3.34
Late-stage gametocyte	11.92
Male gamete exflagellation	50.09
Female gamete formation	> 100

^a^The IC_50_ value indicated the concentration of ferulenol that inhibits 50% of parasite development

### Effects of ferulenol on parasite mitochondrial membrane potential

**Fig. 6:**
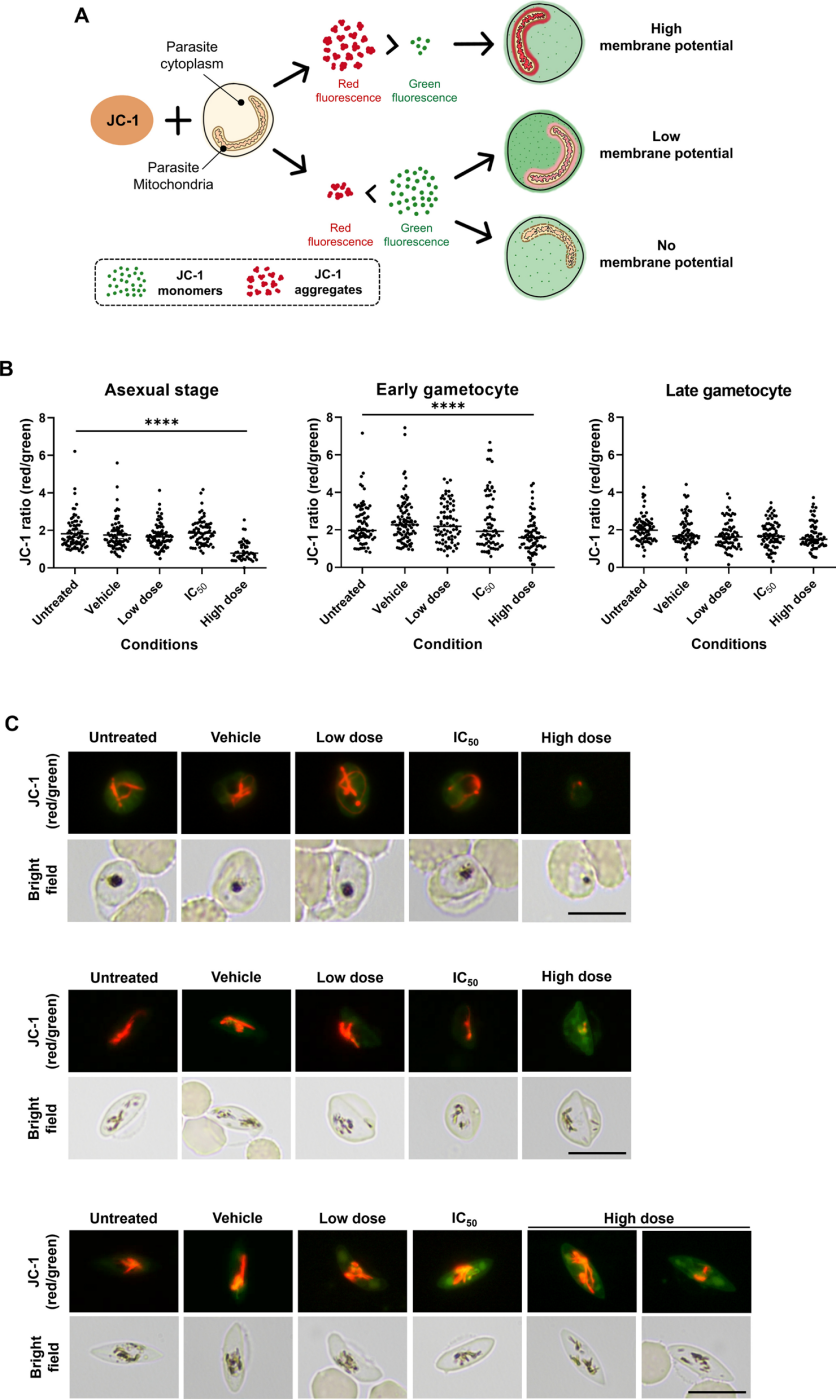
Effects of ferulenol on parasite mitochondrial membrane potential across different developmental stages. **A** Schematic representation of JC-1 staining used to assess mitochondrial membrane potential (ΔΨm). In healthy mitochondria with intact ΔΨm, JC-1 aggregates in the mitochondria and fluoresces red. In mitochondria with a disrupted ΔΨm, JC-1 remains in its monomeric form in the parasite cytoplasm and fluoresced green. Consequently, the JC-1 ratio (red/green) was higher in parasites with normal ΔΨm compared to those with disrupted ΔΨm. **B** A dot plot of > 20 parasites represents individual measurements of JC-1 fluorescence ratio (red/green fluorescence intensity) in parasites (asexual blood-stage, early-stage gametocyte, and late-stage gametocyte) treated with ferulenol at three concentrations: high dose (100 µM), IC_50_, and low dose (1 µM). Horizontal lines indicate the median. Statistical significance was assessed using one-way ANOVA. **C** Representative fluorescent images of the JC-1-stained parasite at each stage are illustrated. The high dose of ferulenol caused a marked reduction in red fluorescence, indicating a loss of ΔΨm in asexual blood stage and early-stage gametocytes. No significant changes were observed in late-stage gametocytes across all treatment groups. Scale bars indicated 10 µm

During electron transport through the mitochondrial membrane, protons (H⁺) accumulate in the intermembrane space of the mitochondrion, generating the mitochondrial membrane potential. To investigate whether ferulenol directly interferes with mitochondrial membrane potential—a critical component of mitochondrial energy metabolism—parasites were treated with ferulenol at three concentrations: a high dose (100 µM), the IC_50,_ and a low dose (1 µM). The IC_50_ values of ferulenol were determined for each parasite stage: 21.67 µM for asexual blood stages, 3.34 µM for early-stage gametocytes, and 11.92 µM for late-stage gametocytes. The treatment was applied according to the protocol for each stage of parasite development. After treatment, the parasites were stained with JC-1 dye to assess mitochondrial membrane potential (Fig. 6A).

The results showed that only the high dose of ferulenol significantly reduced the JC-1 ratio, indicating that only this dose effectively interfered with mitochondrial membrane potential (Fig. 6B, C). However, in the late-stage gametocyte experiment, no significant differences were observed between any of the groups (Fig. 6B, C), suggesting that ferulenol had no observable effect on mitochondrial membrane potential in this stage. These findings imply that the lethality and developmental disruption observed in parasites treated with ferulenol may be due to mechanisms other than direct inhibition of the mitochondrial membrane potential. Moreover, it is important to note that this effect seems more prominent in early asexual stages and early-stage gametocyte development than in late-stage gametocytes.

## Discussion

This study demonstrated the effects of ferulenol on the developmental stages of *P. falciparum*, from asexual stages to gamete formation. It emphasized the potential of ferulenol to both inhibit parasite growth and block transmission. In the asexual blood stage, ferulenol inhibited parasite development at low micromolar concentrations [21]. Although the level of inhibition observed in this study was slightly lower than reported in previous studies, it confirmed the potential of the compound to suppress asexual blood-stage parasite growth. One possible reason for the difference between the findings of this study and those of the previous report was the assays used to quantify the effects. The light microscope-based investigation was possibly less sensitive than the lactate dehydrogenase assay performed in a previous study. However, bright-field microscopy had the advantage of identifying pathological changes in the parasite following drug treatment, which specifically confirmed the drug's effects on parasites.

Ferulenol has been identified as targeting mitochondrial ATP synthesis [22, 29, 30]. However, previous studies suggested that asexual blood stage parasites rely mostly on ATP from glycolysis [6, 7]. This evidence indicated that ferulenol might also affect other biological processes that are critical to parasite survival. Prior research reported that ferulenol targets *Plasmodium* malate; quinone oxidoreductase (*Pf*mqo), an enzyme that directly participates in the TCA cycle and electron transport chain [21, 22, 31, 32]. Furthermore, it contributed to nucleotide synthesis by generating oxaloacetate, a precursor for purine salvage and pyrimidine biosynthesis. Additionally, a study on rodent malaria showed that *Pb*mqo knockout parasites exhibited reduced growth and decreased virulence of cerebral malaria in *Plasmodium berghei* ANKA strain infection [19]. Apart from its effects on *Pf*mqo inhibition, ferulenol has also been reported to target the *bc*_1_ complex [20]. Atovaquone, an approved antimalarial drug that also targets the *bc*_1_ complex, is effective in inhibiting parasite growth [18]. Notably, however, the morphological phenotypes differed between the two compounds: while atovaquone treatment commonly results in pale cytoplasm and accumulation of hemozoin pigment, reflecting impaired protein and nucleic acid synthesis [15], ferulenol-treated parasites exhibited pronounced cytoplasmic vacuolization and nuclear abnormalities. These distinct pathological features suggest that ferulenol may disrupt additional pathways, such as nuclear integrity or oxaloacetate-dependent biosynthesis, beyond the classical *bc*_1_ inhibition observed with atovaquone. The morphological changes of the parasite treated with antimalarial drugs, including artesunate, chloroquine, and methylene blue, also supported the distinctive effect of ferulenol (Supplementary figure).

This study is the first to report the effects of ferulenol on gametocyte development and gamete formation. As hypothesized, ferulenol clearly inhibited gametocyte development. Interestingly, ferulenol predominantly affected early-stage gametocytes, demonstrating a greater impact than that observed in both the asexual stage and late-stage gametocytes. This finding highlighted the essential role of mitochondrial activity in gametocyte and sexual stage development, starting as early as the early-stage gametocyte. Previous studies showed ultrastructural differences of mitochondria between those found in asexual blood-stage parasites and gametocytes [12]. For instance, gametocyte mitochondria exhibited branching patterns and cluster-like shapes, distinct from the single mitochondrion in asexual stages, which branched only at the schizont stage until segregated into merozoites. This structural differentiation suggested increased mitochondrial activity in gametocytes to produce sufficient ATP for complicated biological processes in gametocyte and sexual stage development.

Theoretically, mitochondrial activity increased and became more critical in a stage-dependent manner, as shown in prior studies. Thus, it would be expected that ferulenol could interfere more with late-stage gametocyte development than with early-stage development. Contrary to this expectation, the results clearly showed that ferulenol predominantly affected early-stage gametocyte development. This phenomenon has also been observed in inhibitor-treated late-stage gametocytes. A previous study demonstrated that late-stage gametocytes reduced drug uptake by lowering new permeability pathways (NPP) activity, thereby decreasing drug efficacy against late-stage gametocytes [33].

The long-term ferulenol treatment experiment confirmed the essential role of mitochondrial activity in gametocyte development. This study showed that treating gametocytes with ferulenol from the early stage significantly reduced stage V gametocyte maturation by > 90%. This finding suggested that targeting mitochondria during early-stage gametocyte development was a viable strategy to inhibit progression. Furthermore, this study provided novel insights into ferulenol's potential as a transmission-blocking agent. Nevertheless, whether ferulenol-treated individuals could recover and contribute to transmission was not assessed. Therefore, the potential transmission-blocking effects should be interpreted with caution and warrant further validation. In addition to inhibiting gametocyte development from the early stages, high doses of ferulenol interfered with male gamete formation, particularly compared to female gamete formation. This result suggested that male gametogenesis, including the exflagellation process, was more sensitive to disruptions in mitochondrial activity than female gamete formation. Similarly, previous studies also reported that mitochondria of male gametes were less active but more sensitive to mitochondrial inhibition than those of female gametes [11].

Taken together, these findings suggested that ferulenol shows potential as a dual-action lead compound to target both the asexual blood stage and the sexual stage of the malaria parasite. However, further optimization and toxicity studies are required for safe use in humans [21, 34, 35]. In addition, the experimental design involved continuous drug exposure for up to 72 h, which may raise concerns regarding prolonged drug pressure and the potential selection of resistant parasites. While the primary aim of this study was to investigate stage-dependent effects of ferulenol, rather than to model resistance acquisition, future work should include shorter, fixed-concentration exposures to better evaluate resistance-related risks. Moreover, the narrow selectivity index limits the possibility of ferulenol as a stand-alone antimalarial candidate. Although inhibitory effects were observed at relatively high concentrations, these findings highlight the potential of PfMQO and related pathways as promising druggable targets. Future studies exploring structural optimization or combination therapies could improve potency and reduce the required dose, enhancing translational feasibility. In this context, exploring ferulenol in combination therapies may represent a more promising strategy. Indeed, a previous study demonstrated strong synergism between ferulenol and atovaquone [21], raising the possibility that such combinations could enhance efficacy, mitigate toxicity, and potentially reduce the risk of resistance commonly associated with atovaquone monotherapy.

## Conclusions

This study provided novel insights into the antimalarial potential of ferulenol, demonstrating its dual action on both the asexual blood stage and the sexual stage of *P. falciparum*. Long-term treatment significantly reduced stage V gametocyte maturation, and ferulenol also interfered with male gamete formation, suggesting its potential as a transmission-blocking agent. The observed sensitivity of early-stage gametocytes to ferulenol highlighted the critical role of mitochondria in gametocyte development and sexual stage progression. Although ferulenol showed promise as a dual-action antimalarial lead compound, further studies on its toxicity profile, potential synergism with other drugs, and effect on the drug-resistant strain of parasite were essential. These findings positioned ferulenol as a valuable candidate for the development of new antimalarial therapies targeting both disease severity and transmission.

## Supplementary Information

Supplementary material 1.

## Data Availability

The datasets supporting the conclusions of this article are included within the article and its additional files (supplementary figure).

## Abbreviations

ANOVA: Analysis of variance
ATP: Adenosine triphosphate
CM: Complete medium
DMSO: Dimethyl sulfoxide
GDI: Gametocyte development inhibition
HCT: Hematocrit
hpi: Hours post-infection
IC_50_: Half-maximal inhibitory concentration
ICM: Incomplete medium
JC-1: 5,5′,6,6′-Tetrachloro-1,1′,3,3′-tetraethylbenzimidazolocarbo-cyanine iodide
MQO: Malate: quinone oxidoreductase
NPP: New permeability pathway
PBS: Phosphate-buffered saline
*Pf*mqo: *Plasmodium falciparum* Malate: quinone oxidoreductase
SD: Standard deviation
TCA: Tricarboxylic acid cycle
μM: Micromolar
ΔΨm: Mitochondrial membrane potential
