# Supplementary figures and images for: Disruption of mitochondrial function in blood and sexual stages of Plasmodium falciparum by ferulenol
Source: Parasit Vectors. 2025 Nov 5;18:450. doi: 10.1186/s13071-025-07103-4 (PMC12590880; doi:10.1186/s13071-025-07103-4)

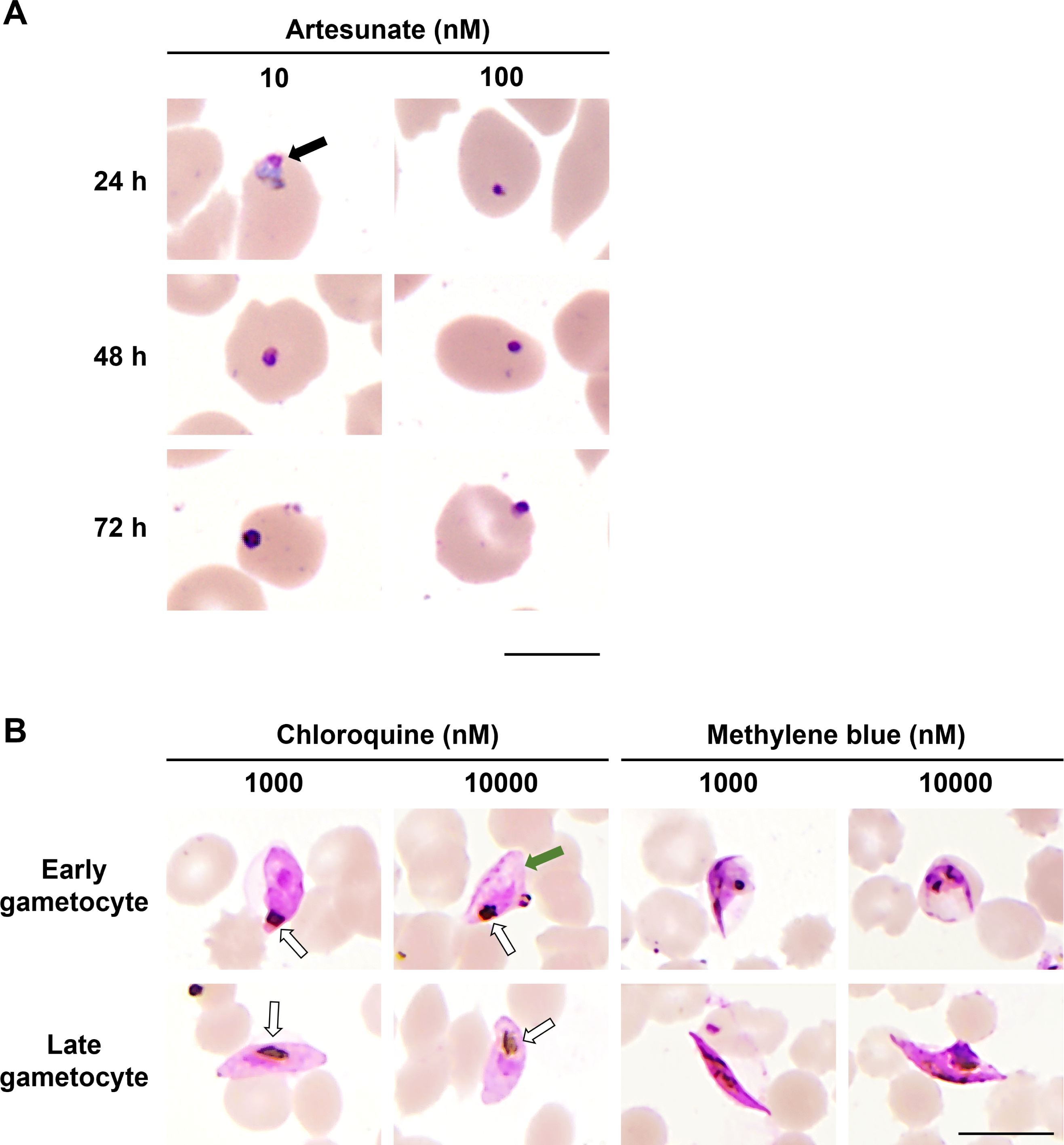

Supplement: Supplementary file 1 — Supplementary material 1. [file 13071_2025_7103_MOESM1_ESM.tif]
